# Ustekinumab and Vedolizumab Dual Biologic Therapy in the Treatment of Crohn's Disease

**DOI:** 10.1155/2017/5264216

**Published:** 2017-11-08

**Authors:** Eddie Y. Liu, Dustin E. Loomes

**Affiliations:** ^1^University of Saskatchewan, Saskatoon, SK, Canada; ^2^University of British Columbia, Vancouver, BC, Canada

## Abstract

We present a case of refractory ileocolonic Crohn's disease in a 27-year-old female treated with dual ustekinumab and vedolizumab biologic therapy. She had mucosal healing for the first time in 13 years after a 10-month treatment of ustekinumab overlapped with 6 months of vedolizumab. No side effects were observed during the 6 months of dual biologic therapy. Short-term dual biologic therapy may be considered as a treatment option for induction of remission in refractory cases of Crohn's disease.

## 1. Introduction

Crohn's disease is an inflammatory condition of the gastrointestinal tract that is associated with significant morbidity [1]. Medical therapies that are commonly used include oral 5-aminosalicylates, antibiotics, conventional glucocorticoids, nonsystemic glucocorticoids, immunomodulators, and biologic therapies. In patients who do not respond to medical therapy, surgical resection can lead to clinical remission. However, surgical resection is not curative and most patients eventually relapse [2].

## 2. Case Presentation

We present a case of refractory ileocolonic Crohn's disease in a 27-year-old female treated with dual ustekinumab and vedolizumab biologic therapy. She was diagnosed at age 14 and underwent a right hemicolectomy at age 17, followed by small bowel resections at age 20 and 21 for medically refractory disease. She was previously treated with azathioprine, prednisone, infliximab, adalimumab, and ustekinumab as well as clinical trials involving tofacitinib and mongerson.

**Figure 1:**
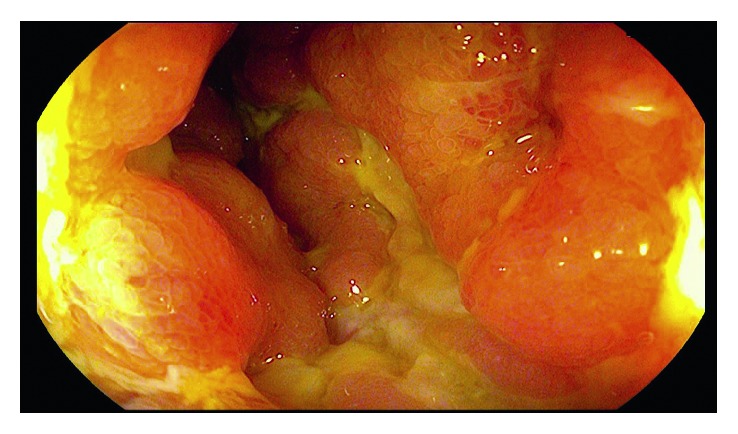
Colonoscopy showing severe ileal serpiginous ulceration and diffuse ileitis (Rutgeerts classification i4) before starting vedolizumab and ustekinumab therapy.

**Figure 2:**
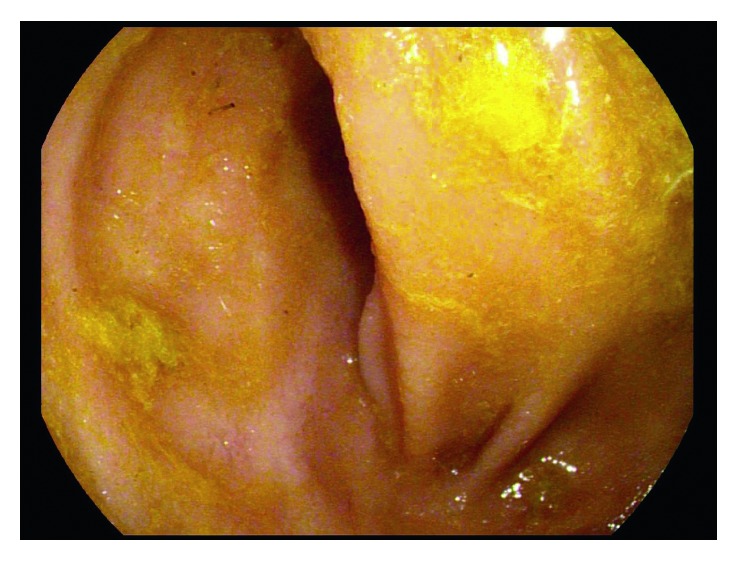
Colonoscopy showing ileal mucosal healing 6 months after starting vedolizumab and ustekinumab therapy.

She was assessed for ongoing severe Crohn's symptoms and iron deficiency anemia after receiving a retrial of infliximab with the addition of methotrexate combination therapy. Colonoscopy showed ongoing deep serpiginous ileal ulcers, colonic aphthous ulceration, and worsening deep rectal ulceration (Figure 1). As her previous ustekinumab therapy had not included intravenous loading, infliximab combo therapy was stopped and she was restarted on ustekinumab with induction dosing and then 90 mg subcutaneously every 4 weeks, in combination with azathioprine. Azathioprine metabolites showed significant shunting with a low 6-thioguanine and an elevated 6-methyl mercaptopurine, prompting a dose reduction to 25 mg daily and addition of allopurinol 50 mg daily. Mezavant 4 g daily, salofalk 1 g suppositories, and Entocort 9 mg daily were also started. Despite this, symptoms continued, with magnetic resonance enterography and fecal calprotectin (FCP) both showing ongoing evidence of inflammation five months post initiation of ustekinumab. After careful consideration and discussion of known and unknown risks of dual biologic therapy, vedolizumab was added, and ustekinumab was decreased to every 8 weeks. Five months after adding vedolizumab to ustekinumab, FCP decreased to 93 mcg per gram, and abdominal pain and nausea began to improve. Colonoscopy 6 months after dual biologic therapy showed mucosal healing of the ileum and colon (Figure 2), which is the first time the patient has had mucosal healing since diagnosis. No side effects from her medical therapy have arisen thus far. Repeat azathioprine metabolites targeting a 6-thioguanine of 125 pmol/8 × 10^8^ RBC are pending, and we plan to obtain an ustekinumab level and stop dual biologic therapy, continuing with vedolizumab and azathioprine combination therapy.

## 3. Discussion

This case demonstrates the benefit of using dual biologic therapy with ustekinumab and vedolizumab in treating severe ileocolonic Crohn's disease. Brief addition of vedolizumab during infliximab maintenance therapy has been described in treating ileocolonic Crohn's disease [3]. There is a paucity of data on the safety of dual biologics. In a small case series of 3 patients given 2-3 doses of ustekinumab during anti-TNF therapy, no adverse effects in a median follow-up of 21 months emerged [4]. Additionally, no adverse effects arose after 10 months of vedolizumab and etanercept dual therapy in treating chronic pouchitis and spondyloarthritis [5]. Although short-term dual biologic therapy may be considered as a treatment option for induction of remission in refractory cases of Crohn's disease, larger studies are needed to assess the efficacy and safety of this approach.
